# Production and Heat Properties of an X-ray Reflective Anode Based on a Diamond Heat Buffer Layer

**DOI:** 10.3390/ma13010241

**Published:** 2020-01-06

**Authors:** Xinwei Li, Xin Wang, Ye Li, Yanyang Liu

**Affiliations:** School of Science, Changchun University of Science and Technology (CUST), Changchun 130022, China; lixinweiwill@163.com (X.L.); liye@cust.edu.cn (Y.L.); liuyanyang@cust.edu.cn (Y.L.)

**Keywords:** diamond, heat buffer layer, micro-focus, thermal stability

## Abstract

This paper introduces an X-ray reflective anode with a diamond heat buffer layer, so as to improve heat dissipation of micro-focus X-ray sources. This also aids in avoiding the destruction of the anode target surface caused by the accumulation of heat generated by the electron beam bombardment in the focal spot area. In addition to the description of the production process of the new reflective anode, this study focuses more on the research of the thermal conductivity and compounding ability. This paper also introduces a method that combines finite element analysis (FEA) in conjunction with thermal conductivity experiments, and subsequently demonstrates the credibility of this method. It was found that due to diamonds having a high thermal conductivity and melting point, high heat flux produced in the micro-focus spot region of the anode could be conducted and removed rapidly, which ensured the thermal stability of the anode. Experiments with the power parameters of the radiation source were also completed and showed an improvement in the power limit twice that of the original.

## 1. Introduction

In the field of X-ray imaging, the improvement of image resolution has always been a key point for research. According to the projection imaging principle, there is an inverse relationship [1,2,3] between the size of the focal spot of the X-ray source and the space resolution of the images. In other words, the smaller the focal spot is, the higher the space resolution will be, and vice versa. Therefore, the improvement of the space resolution through the reduction of focal spot size is an important research subject. However, a smaller focal spot means higher energy from the electron beam is hitting the metal target, which means a need for more efficient cooling [4]. Therefore, transmission targets which could be cooled at end-windows were adopted for high resolution applications of X-ray imaging because they could fix this cooling problem. However, these targets suffer from low X-ray transmission; therefore, transmission X-ray sources are inferior to reflective sources in X-ray generation efficiency. Regardless, reflective sources still suffer from the problem of heat dissipation [5] and the reflective anodes have effective focal spots smaller than the actual spots. However, in order to take advantage of the high efficiency of reflective X-ray anodes, as well as to solve the problem of heat dissipation, rotation anodes [6,7], liquid-metal anodes [8,9] and water cooled anodes [10] were invented. However, they were also accompanied by anode vibration and structural complexity, which resulted in complicated computing for image analysis and therefore limited the applications of the inventions. Essentially, it is advantageous to maximize X-ray intensity to increase signal-to-noise ratio and minimize analysis complexity but preventing damage to the anode from excessive heating is usually the limiting factor [11].

Based on the high thermal conductivity (TC = 1500–2000 W/m K) [12,13,14] of diamond, materials containing diamond are widely used in high-power microelectronic devices as a heat dissipator [15,16]. In order to improve the thermal conductivity of composite materials, a method for mixing diamond particles with a metal, and a method for plating a diamond film were proposed [17,18]. However, in this study, the C–C structure of diamond cannot excite higher-intensity X-rays, so it cannot directly serve as an anode. Moreover, its chemical properties are stable, meaning it is hard to react with the copper substratum. Therefore, the study aims at a method for preparing reflective X-ray anodes utilizing diamond as heat buffer layer (diamond composite anodes). Furthermore, the diamond composite anode’s major properties were determined for the purpose of broadening the effective application range.

## 2. Experiment

### 2.1. Production Process of the Diamond Composite Anode

**Figure 1:**
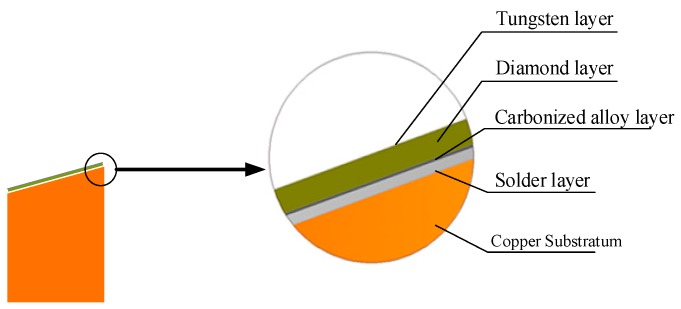
Structural diagram of the X-ray composite anode.

Figure 1 shows the composition of the diamond composite anode, which was produced through the following steps and methods.

(1) Preparation of the Diamond Heat Buffer layer

Used for uniform homogeneity, low deposition temperatures, and slight influences on physical properties of the diamond, in this experiment, the PECVD [19] (plasma enhanced chemical vapor deposition) method is most suitable to produce the thermally conducting diamond layer. In this paper, the diamond heat buffer layer was prepared on a Mo slice as per the PECVD method.

**Figure 2:**
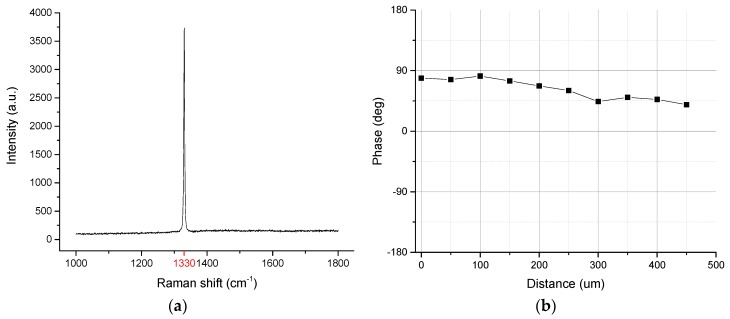
(**a**) Raman spectrum of the diamond film; (**b**) thermal diffusion coefficient of diamond film.

Figure 2a shows the Raman spectrum of a diamond film. In the figure, a characteristic absorption peak for diamond exists at 1330 cm^−1^, and is an absence of absorption peaks for other non-diamond carbon structures. Figure 2b shows the thermal diffusion coefficient of diamond measured with photo-thermal deflection (PTD) thin-film thermal conductivity test system [20]. Thermal conductivity was derived from the measured thermal diffusion coefficient. Therefore, the diamond layer parameter in this study included a thickness of 0.5 mm, a thermal conductivity of 1893 W/m K, and a thermal stress coefficient of 1.2 × 10−6/°C.

(2) Surface Metallization of the Diamond Heat Buffer layer

The diamond heat buffer layer separated from the Mo slice as soon as it was taken off the PECVD instrument after preparation. Then the diamond sheet was bonded tightly to a copper substratum. It was difficult to weld the diamond heat buffer layer and the copper substratum together by conventional soldering or plating because of the poor wettability and reactivity [21] between them. Therefore, metallization on the diamond surface was necessary, and Ti was taken as the metallization material. The metallization procedure consisted of a vacuum-evaporation coating process, which left a film of Ti on the diamond surface, and a high temperature annealing process during which Ti reacted with the diamond surface and produced a TiC structure that had a stable Ohmic contact with the diamond surface. Therefore, the diamond surface became highly adhesive [22,23].

(3) Soldering the Copper Substratum and the Diamond Heat Buffer layer

A special proportion of solder alloy was used under vacuum within high temperature conditions to solder the metallized diamond heat buffer layer and the copper substratum together, leaving a 15-μm-thick solder layer. Melt solder, wetting both the copper surface and the diamond surface, was cooled down. A gradient temperature was adopted for the heating and cooling to prevent the solder layer from cracking and loosening which can result from the different thermal expansion coefficients of the materials.

(4) Metal Plating (Tungsten) on the Other Diamond Surface

After the soldering was completed, the next step in the production process was to apply a tungsten film on the other surface of the diamond sheet. Tungsten, with a high melting point, is used widely in X-ray anode production. In addition, magnetron sputtering, a good method to extensively deposit high quality films, was utilized for the surface treatment on the other side of the X-ray anode. A general tungsten slice was used as a target in the experiment and a 2.2-μm-thick tungsten film was deposited on the other surface of the diamond sheet.

### 2.2. Property Test of the Diamond Composite Anode

In this study, tests were conducted for determination of the key properties of the diamond composite anode produced, which included:

(1) Test of Bonding Strength between Layers

It is necessary to test the inter-layer bonding strength, which may influence the diamond composite anode’s scattering and other properties. The test was completed directly by cross-section characterization and analysis. The layers consist of the tungsten film, the diamond heat buffer layer, the metallization layer, the solder layer and the copper substratum. Scanning electron microscopy (SEM) [24] was used to observe the inter-layer smoothness and coverage.

(2) Test of Bulk Temperature in the Focal Spot Region

A multi-functional X-ray generator, which is capable of simulating various actual work conditions of X-ray sources such as power and focal spot size etc., was used to determine and compare the bulk temperature in the focal spot region for different kinds of anodes. Under the conditions of constant power, the generator successfully kept the focal spot in a circular shape and at a size of 20 μm, which is the reference standard spot size most widely used in actual micro-focus X-ray inspections. The diamond composite anode was compared with a conventional tungsten film anode in the bulk temperature in focal spot region.

a. Test of Surface Temperature in Focal Spot Region

At present, there is no efficient way to detect surface temperatures in focal spots of X-ray anodes. Therefore, the proposal in this paper was to measure the infrared radiation spectrum at the focal spot and indirectly calculate the temperature in the spot by combining spectral analysis and processing technology. This will be explained specifically in the following section:

For focal spot temperature determination, it was difficult to precisely locate the X-ray focal spot on an anode because the spot size was near to that of the infrared detector’s individual pixel. Therefore, infrared imaging spectrographs capable of detecting wave bands in the range 900–2500 nm, as well as an infrared lens whose focus length was 300 mm, were adopted in this study to scan the anodes inside the X-ray generator and obtain the infrared images in which each pixel had a corresponding infrared spectral curve. That is, through analysis and calculation on the infrared spectral curves corresponding to effective pixels projected by a focal spot on the CCD (Charge Coupled Device) the temperature at a specific location of the focal spot was identified.

b. Comparison of the Vertical Temperature Distribution of Each Layer in the Focal Spot Region

The diamond composite anode is a reflective X-ray source, whose heat is mainly conducted in the vertical direction, but the infrared imaging spectrographic method mentioned above can only measure the temperature on the surface of tungsten film and cannot characterize the anode’s vertical temperature distribution (in a direction vertical to the surface of the anode). A super non-uniform vertical temperature distribution can cause stress or structure changes in the materials of the anode and then degrade the inter-layer bonding strength. At present, it is still impossible to determine the vertical heat distribution in the focal spot region of the micro-focus X-ray anode through experimental methods. Therefore, the finite element method (FEM), which is usually used in heat transfer analysis, was introduced in this study to determine the vertical temperature distribution of each layer in the focal spot region. FEM can solve the position data for the related nodes inside the complex anode through discrete processing using temperature and heat flux. This method can characterize the temperature and heat flux density trends from the surface of the focal spot to the surface of the copper substratum. Moreover, the ANSYS software program (based on finite element analysis) [25,26] used in this experiment is very suitable for the thermal conductivity analysis of composite materials. Thus, it was used to model the composite anode. However, the choice of the ANSYS is also based on the properties of the material, the composite method, and the heat transfer process.

**Figure 3:**
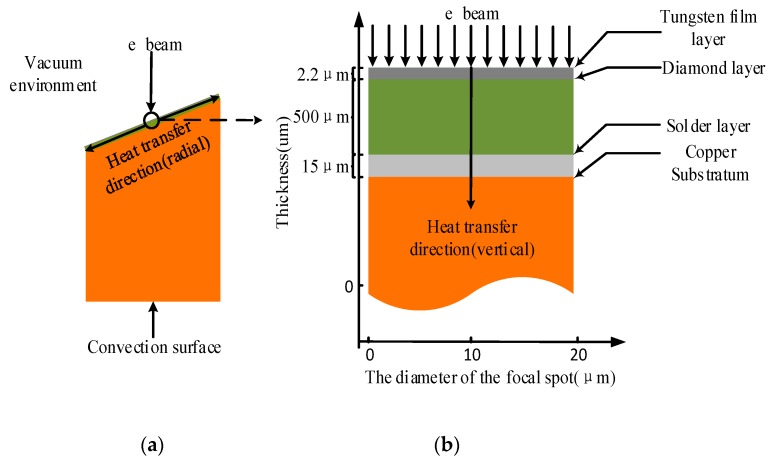
Two-dimensional unsteady heat transfer model of the composite anode. (**a**) Overall scheme of heat transfer of the diamond composite anode; (**b**) scheme of vertical heat transfer within the focal spot region.

According to experimental conditions and the properties of the materials constituting the diamond composite anode, the heat transmission experimental procedure can be turned into a two-dimensional unsteady heat transfer model, as shown in Figure 3a. This was due to the requirement of establishing a systematical heat transfer model before conducting finite element analysis. Figure 3b shows the magnified cross-section of the diamond composite anode at the focal spot region. Due to the diameter of the electron beam spot being far smaller than that of the composite anode, the analysis was approximated to the area where the electron beam bombardment was actually on the tungsten film. Therefore, one assumption was that this smaller specific area heated up evenly. Therefore, the heat generated by the electron beam bombardment transferred to the interior of the diamond composite anode evenly.

Direction of Anode Internal Heat Transfer: the horizontal (surface) heat transfer refers to the thermal conduction from the center of the focal spot to edge of the anode, while vertical inter-layer heat transfer refers to that through layers of the diamond composite anode to its convection surface, which depends on natural cooling. Then, the finite element method was used to determine the surface temperature and the vertical heat distribution at the focal spot of the diamond composite anode by giving a particular initial and a boundary temperature value. Similarly, the vertical heat distribution of each layer in the focal spot region of three other kinds of anodes (conventional tungsten anodes, tungsten film anodes, and anodes with silver heat buffer layer) were analyzed and compared.

c. Comparison of Temperatures at the Power Limit

The power was adjusted but other parameters of the X-ray generator remained unchanged to compare the maximum temperature distribution in each layer of the four types of anodes. The procedure mentioned above was followed for determination of the vertical and horizontal heat transfer of X-ray anodes at the focal spots.

## 3. Results and Discussion

### 3.1. Results of and Discussion on Inter-Layer Bonding Strength Tests

**Figure 4:**
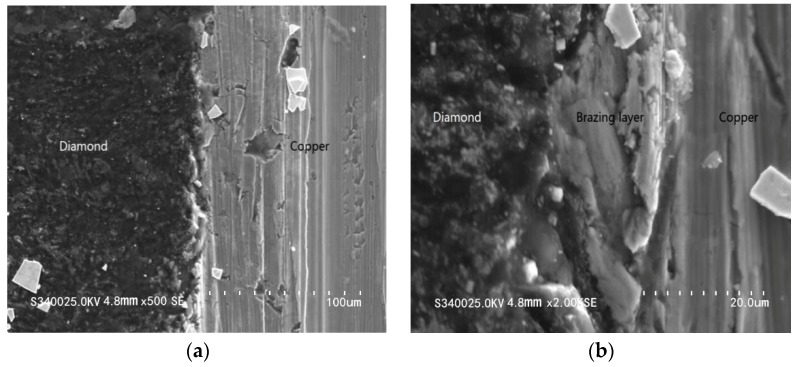
Cross-sectional images of the composite anode under scanning electron microscopy (SEM). (**a**) Cross-sectional image of the soldering layer, magnified 500 times; (**b**) cross-sectional image of the soldering layer, magnified 2000 times.

In Figure 4, the cross-sectional images of the composite anodes show the bonding results between layers after the interfacial reaction, observed by a SEM. Figure 4a is the cross-sectional image of the diamond composite anode, magnified 500 times, in which the brazing position shows smoothing and no obvious gap or crack can be identified in the diamond heat buffer layer (dark black) or the copper substratum (light grey). Figure 4b is the cross-sectional image of the diamond composite anode’s soldering layer, magnified 2000 times, in which no obvious splits can be identified in the diamond heat buffer layer or the copper substratum. There is no obvious separation between the solder layer and the diamond heat buffer layer. It can be concluded that there was wetting of diamond heat buffer layer, solder, and copper substratum.

### 3.2. Results of and Discussion on Temperature Measurements at Focal Spot Region

#### 3.2.1. Surface Temperatures at Focal Spot Region

The scanning distance across the anode surface was completed by an iterative approach where the distance was reduced approaching the focal spot region. In this way, surface temperature measurements of the anode reduced interference and improved measurement precision. The focal spot projected two effective pixels on the CCD, and the number was sufficient for temperature measurements at the anode’s focal spot. Then, by repeating measurements and calculations, the relationship between time (duration of electron beam bombardment) and surface temperature at the focal spot region was determined.

**Figure 5:**
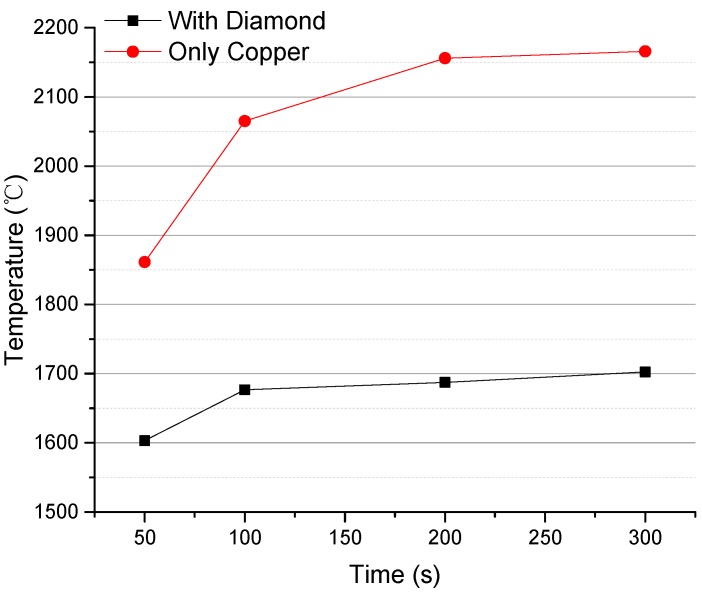
Diamond composite anode vs. common tungsten film anode in surface temperature under the same conditions.

Figure 5 shows the variation trend over time for the composite anode’s surface temperature. The surface temperature of the anode with a diamond buffer was always much lower than that of a tungsten film anode, at any time. For example, at 50 s, the surface temperature difference was 258 °C, and at 300 s, the surface temperature difference was 463 °C. Hence, the new X-ray reflective anode with the diamond heat buffer layer introduced in this study is a breakthrough in research for heat dissipation in a focal spot region of a micro-focus X-ray anode.

#### 3.2.2. Comparison of Vertical Temperature Distribution of Each Layer in the Focal Spot Region

The reliability of the finite element method was verified by surface temperature measurements in the focal spot region. This was completed by comparing the temperature value obtained using the method with that determined through experimental measurement.

**Figure 6:**
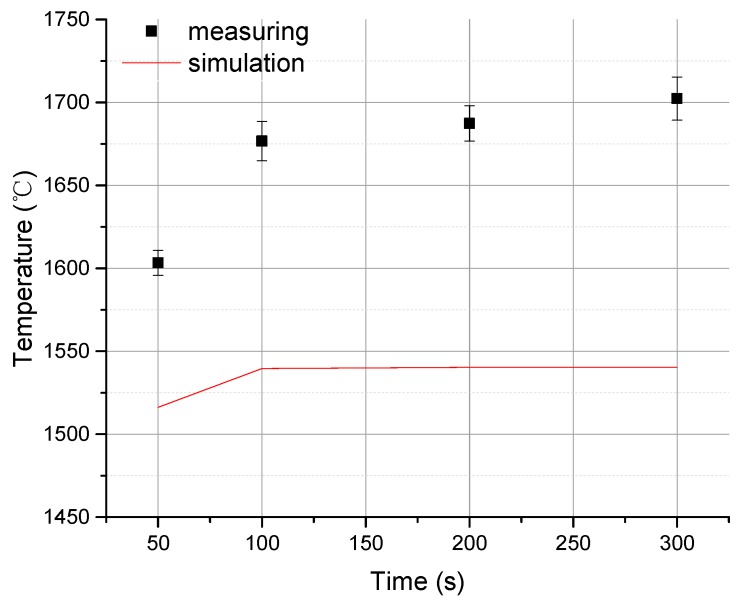
Measured value vs. simulated value in surface temperature in the focal spot region of the diamond composite anode.

Figure 6 shows the anode surface temperature values obtained at different times by modeling and simulation using the finite element method, as well as the mean of triplicate experimental measurements. In Figure 6, the point represents the change of the maximum temperature value of the measured focus position with time. The straight line represents the change of the maximum temperature value of the simulation focus position with time. It also indicates the maximum temperature change process when the focus changes from non-thermal equilibrium to thermal equilibrium.

Surface temperature values obtained at different times by modeling and simulation and by experimental measurements are close and the average error does not exceed 9.2%. Hence, the finite element method used in this study is reliable. However, there are still two facts which can decrease the reliability: first, the simulation assumes an absolute vacuum, however in reality there is always a certain amount of oxygen trapped in the vacuum chamber which may react with the anode’s tungsten film and produce an oxide; and second, the soldering alloy used may not have uniform composition or contain impurities, resulting in slight value errors through the determination of the thermal conductivity and heat flux.

**Figure 7:**
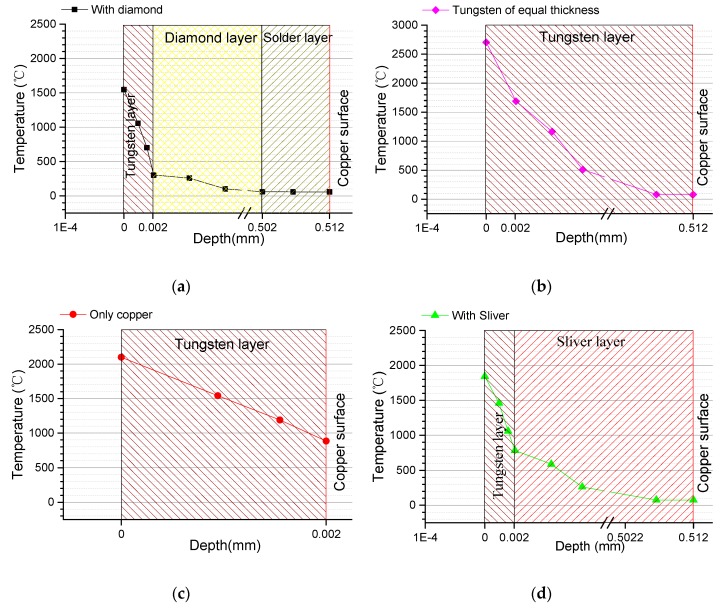
Comparison of vertical heat distributions at the focal spots between four types of anodes: (**a**) composite anode, (**b**) conventional tungsten anode, (**c**) tungsten film anode and (**d**) anode with silver heat buffer layer.

To prove the superiority of the new diamond buffer layer anode in all conditions, two aspects, the temperature and heat flux of each layer, were analyzed. Figure 7 shows the highest temperature value at the focal spots in each layer of the four types of compared anodes. Temperature comparisons between type (a) and type (b) demonstrate that the composite layer (the part above the surface of the copper substratum) of type (a) is as thick as the entire tungsten plate of type (b). As shown in Figure 7b, the temperature on the surface of the anode was different from that on the surface of the copper substratum. This results in the temperature on the surface of the anode at the focal spot quickly reaching the melting point of tungsten, were even at a low power for X-ray generation. Comparisons between type (a) and type (c) indicate that type (c) contains a much thinner tungsten film which allowed for heat transfer to the copper substratum rapidly, but too much heat transfer into the copper substratum will melt the copper substratum at the focal spot even if the tungsten film remains solid. Comparisons between type (a) and type (d) show that type (d) contained silver with the highest thermal conductivity as its heat buffer layer. Under the same conditions, type (d) performed similarly in its response to variation in temperature to type (a), but since silver has a low melting point it will break down much sooner than type (a) as the power increases. Damage to the anodes occurs mainly because the excessive electron beam energy concentrates at the focal spot in the tungsten film. Therefore, the heat flux per unit area for the focal spot region becomes higher at a node in the vertical heat distribution. This can be further verified by calculating the heat flux distribution of each layer.

**Figure 8:**
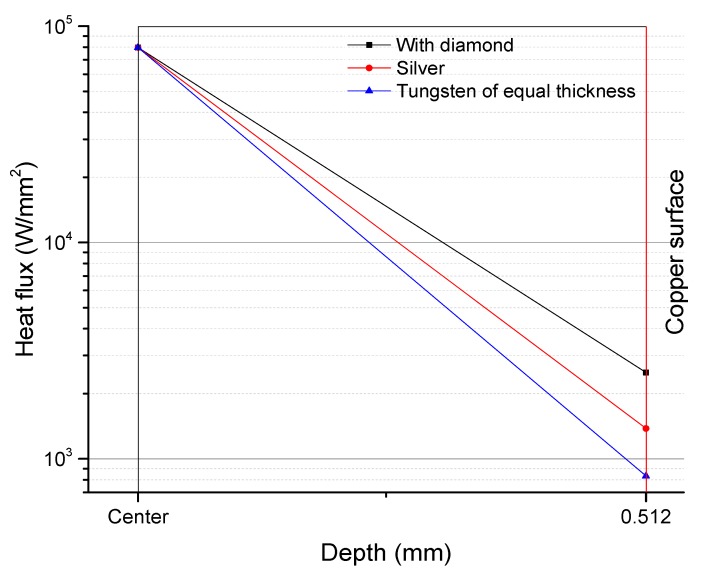
Variations in the heat flux of anodes: type (a) is black line; type (c) is blue line; type (d) is red line.

Figure 8 shows the vertical distribution of heat flux from the tungsten film surface to the surface of the copper substratum of the anodes types (a), (b) and (d). The heat flux on the surface of the copper substratum of type (a) was higher than those of types (d) and (b). Here is the relation between the heat flux and the thermal conductivity:

q = λ(T1 − T2)/δ(1)

The equation is exclusively for one-dimensional vertical direction, where, q refers to the heat flux, T1 refers to the temperature at the center of the focal spot on the tungsten film, T2 refers to the temperature at the center on the surface of the copper substratum, λ refers to the thermal conductivity of the material, and δ refers to the thickness of the material. The equation indicates that when the temperature difference is similar and the thickness of the material is equal, the heat flux q, increases with the thermal conductivity λ. A larger q means there is more heat transferred from the tungsten film to the copper substratum and more heat is conducted away from the unit area. This is why this paper discusses nothing on changes of the heat flux onto the copper substratum of type (c). The tungsten film is thin and the heat flux onto the copper substratum barely changes.

To summarize, the advantage of the new type of anode with a diamond heat buffer layer is that the bulk temperature in the focal spot region of the tungsten film and the temperature on the surface of the copper substratum are both much lower than those of the other types of anodes. This is because diamond is a kind of isotropic material with a polycrystalline structure, and its high interatomic bonding energy gives it a high melting point and a high thermal conductivity. Therefore, the diamond composite anode can rapidly dissipate heat transferred from the tungsten film vertically and horizontally and therefore prevent excessive heat buildup in the tungsten film.

#### 3.2.3. Comparison on Power Limit Temperatures

**Figure 9:**
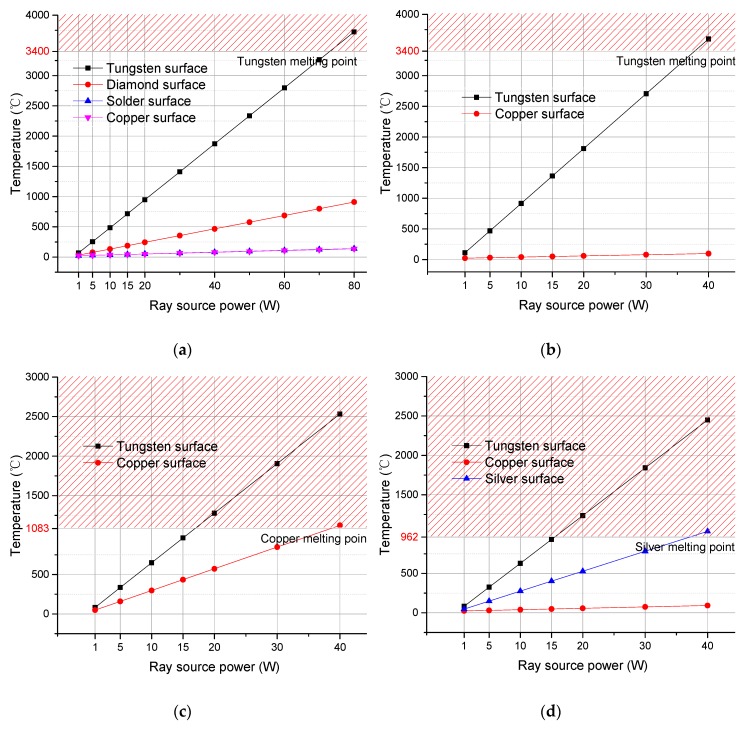
Maximum temperatures in each layer at different powers for four types of anodes: (**a**) diamond composite anode; (**b**) conventional tungsten anode; (**c**) tungsten film anode; (**d**) anode with silver heat buffer layer.

With the increase in the X-ray generator power, the temperature in each layer of an anode increases. Figure 9 shows how the highest temperature in each layer of the 4 types of anodes varies with the power. The power at which the temperature at the focal spot on the surface of an anode reaches the melting point of tungsten is the power limit of the anode. Comparisons between type (a) and type (b) shows that the composite anode has a power limit of 73 W, and the conventional tungsten anode has a power limit of 37.9 W. Comparisons between type (a) and type (c) indicate that when the copper layer is the first to melts, the power limit of type (c) is 38.5 W. Comparisons between type (a) and type (d) show that when the silver layer melts first, the power limit of type (c) is 37.2 W.

It can be seen in Figure 9 that the four types of anodes are similar to each other in the temperature variation tendency for each layer, but only the diamond composite anode had a power limit higher than 40 W. In addition, the diamond composite anode’s power limit was almost twice that of the other three types. Hence, this proves that high thermal conductivity and the high melting point of the diamond composite are advantages.

## 4. Conclusions

A new reflective tungsten X-ray anode with a diamond heat buffer layer was designed, and its inter-layer bonding strength, focal spot temperature limit and thermal stability were examined theoretically and experimentally. This composite anode improves the micro-focus area cooling environment by utilizing the high thermal conductivity of diamond material. Thus, the highly concentrated heat in the micro-focus area is buffered by diffusion and rapid transmission. The conclusions are as follows:

(1) The inter-layer bonding results of the composite anode were smooth, and no obvious gap or crack was found in each layer. This showed that the diamond buffer layer and copper substratum can be combined by brazing.
(2) For the problem of precise temperature measurement at the focal spot on the surface of an X-ray anode, an analytical measurement and processing method based on infrared radiation spectra was proposed in this paper. The highest temperature value corresponding to the spectral peak of the micro-focus area was calculated by the infrared radiation spectrum which measured in the relevant area indirectly. This method was specifically designed for small-focus area temperature measurements.
(3) For the vertical temperature measurement through the cross section of an X-ray composite anode, a method based on finite element was presented in this paper, which was proven reliable by verifying the simulation result with experimental measurements that resulted in surface temperature determination of an anode.
(4) Analysis of heat distribution at the focal spot region of four types of X-ray anodes with different structures demonstrated that the X-ray reflective anode with a diamond heat buffer layer has much higher thermal stability and its working power limit is twice that of other anodes.
